# Multiplexed detection and isolation of viable low-frequency cytokine-secreting human B cells using cytokine secretion assay and flow cytometry (CSA-Flow)

**DOI:** 10.1038/s41598-020-71750-z

**Published:** 2020-09-09

**Authors:** Ayman Rezk, Rui Li, Amit Bar-Or

**Affiliations:** 1 Center for Neuroinflammation and Experimental Therapeutics, Department of Neurology, Perelman School of Medicine, University of Pennsylvania, Philadelphia, PA USA; 2 Neuroimmunology Unit, Department of Neurology and Neurosurgery, Montreal Neurological Institute, McGill University, Montreal, QC Canada; 3 Division of Neurology, Children’s Hospital of Philadelphia, Perelman School of Medicine, University of Pennsylvania, Philadelphia, PA USA

**Keywords:** Cytokines, Lymphocytes, Immunological techniques, Isolation, separation and purification, Biological techniques, Immunology

## Abstract

The ability to functionally characterize cytokine-secreting immune cells has broad implications in both health and a range of immune-mediated and auto-immune diseases. Low-frequency cytokine-defined immune-cell subsets can play key immune-regulatory roles, yet their detailed study is often hampered by limited clinical sample availability. Commonly used techniques including intracellular cytokine staining require cell fixation, precluding subsequent functional interrogation. The cytokine-secretion assay (CSA) can overcome this limitation, though has mostly been used for detection of relatively high-frequency, single-cytokine secreting cells. We examined how adaptation of the CSA in combination with multiparametric flow-cytometry (CSA-Flow) may enable simultaneous isolation of multiple, low-frequency, cytokine-secreting cells. Focusing on human B cells (traditionally recognized as harder to assay than T cells), we show that single-capture CSA-Flow allows for isolation of highly-purified populations of both low-frequency (IL-10^+^; GM-CSF^+^) and high-frequency (TNF^+^) cytokine-defined B cells. Simultaneous detection and isolation of up to three viable and highly-purified cytokine-secreting B-cell subpopulations is feasible, albeit with some signal loss, with fractions subsequently amenable to gene expression analysis and in vitro cell culture. This multiplexing CSA-Flow approach will be of interest in many human cellular immunology contexts aiming to functionally characterize cytokine-secreting immune cells, especially when sample volumes and cell numbers are limited.

## Introduction

Recent technological advances have enabled detailed examination of cells at single-cell resolution, which has served to extend as well as gain novel insights into both phenotypic and functional profiles of different cell types^1, 2^. In the field of immunology, tools such as single-cell RNA sequencing (scRNA-Seq), ATAC-Seq and others continue to reveal previously unrecognized cellular functional heterogeneity, of relevance to both health and a variety of diseases^3–9^. Functional heterogeneity is also reflected in different cytokine expression profiles of immune cells, such as T cells, B cells, innate lymphoid cells and myeloid cells^10–14^. Indeed, particular cytokines have historically been used to define functionally distinct immune cell sub-populations^11,12^ and there is substantial interest in furthering the study of cytokine-expressing immune cells, including their mechanisms of differentiation, maintenance and plasticity^11,12, 14–16^. Such knowledge would have important therapeutic implications across multiple immune-mediated conditions.

Since cytokine expression is context- and activation-dependent^10–14^, most meaningful study of cytokine-defined immune cells relies on isolation of viable cells that are amenable to activation. Indeed, the isolation of highly purified and viable cytokine-defined immune cells is essential for robust interrogation of individual cells such as by scRNA-Seq^17^, and is also required for other downstream applications such as the assessment of the impact of such cells on responses of other cells in vitro, or when introduced in vivo (potentially in the form of adoptive cell transfer^18^). However, the isolation of viable and purified cytokine-expressing immune cells has presented a technical challenge, as commonly used intracellular cytokine-staining (ICS) or flow-fluorescent in-situ hybridization approaches involve cell fixation and permeabilization^19–25^. The resultant loss of cell viability and integrity seriously limits the use of cells for comprehensive functional profiling and downstream applications^19–25^. While viable cells can be sorted based on certain cell surface-markers that have been reported to enrich for particular cytokine-expressing immune cells, such markers are inevitably neither fully sensitive nor specific^26–33^.

**Figure 1:**
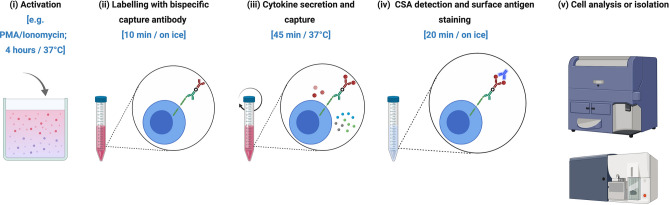
Workflow for the cytokine secretion assay combined with flow cytometry (CSA-Flow). (**i**) Cell activation can be achieved using a polyclonal stimulus (e.g. PMA and ionomycin for 4 h) or an antigen (time-course dependent). (**ii**) Cell suspension are tagged with a bispecific capture antibody that binds to CD45 on the cell surface for 10 min on ice. (**iii**) Cells are incubated in a continuous motion (e.g. rotation) at 37 °C for 45 min to allow for the secretion and capture of cytokine on the surface of cytokine-secreting cells. (**iv**) Cells are counter-stained with a fluorochrome-conjugated anti-cytokine antibody in addition to cell surface labelling for 20 min on ice. (**v**) Cell suspension are analyzed on a flow cytometer or cytokine-secreting cells are FACS-isolated for downstream applications. Figure created with Biorender (biorender.com).

The cytokine-secretion assay (herein referred to as CSA) was designed to overcome this limitation and to allow for the isolation of viable cytokine-defined immune cells^34–36^. CSA relies on bispecific antibodies (bsAbs) bound to the cell surface that capture secreted cytokines on viable cells, thereby preserving cell viability and integrity, in stark contrast to ICS. In essence, CSA typically involves four steps applied to the mixed population of cells: (1) activation (e.g. polyclonal or antigen-specific); (2) labelling with bsAbs that bind on one hand to a cell surface antigen (e.g. CD45 for immune cells) and on the other hand to the particular cytokine of interest; (3) secretion and capture of the cytokine and (4) detection of the cytokine-secreting cells with fluorochrome-conjugated anti-cytokine antibody for flow cytometry analysis (Fig. 1). However, cytokine secretion assays have more typically been applied to capture high-frequency cells^37–39^. In particular, the assay has not been used for simultaneous isolation of multiple low-frequency cytokine-secreting cells, which would be beneficial in many human cellular and immunology contexts, especially when sample volumes and cell numbers are limited.

In this study, we demonstrate the utility of the CSA-Flow for the detection and isolation of viable human cytokine-secreting cells including simultaneous sorting of both low- and high-frequency populations. To illustrate this approach, we focus our attention on human B cells as a growing body of work implicates cytokine-secretion by B cells as relevant in orchestrating immune responses in both health (e.g. host-protection from infection) and different autoimmune diseases (recently reviewed in^12, 40^).

## Results

### Detection of low-frequency cytokine-secreting human B cells in vitro

**Figure 2:**
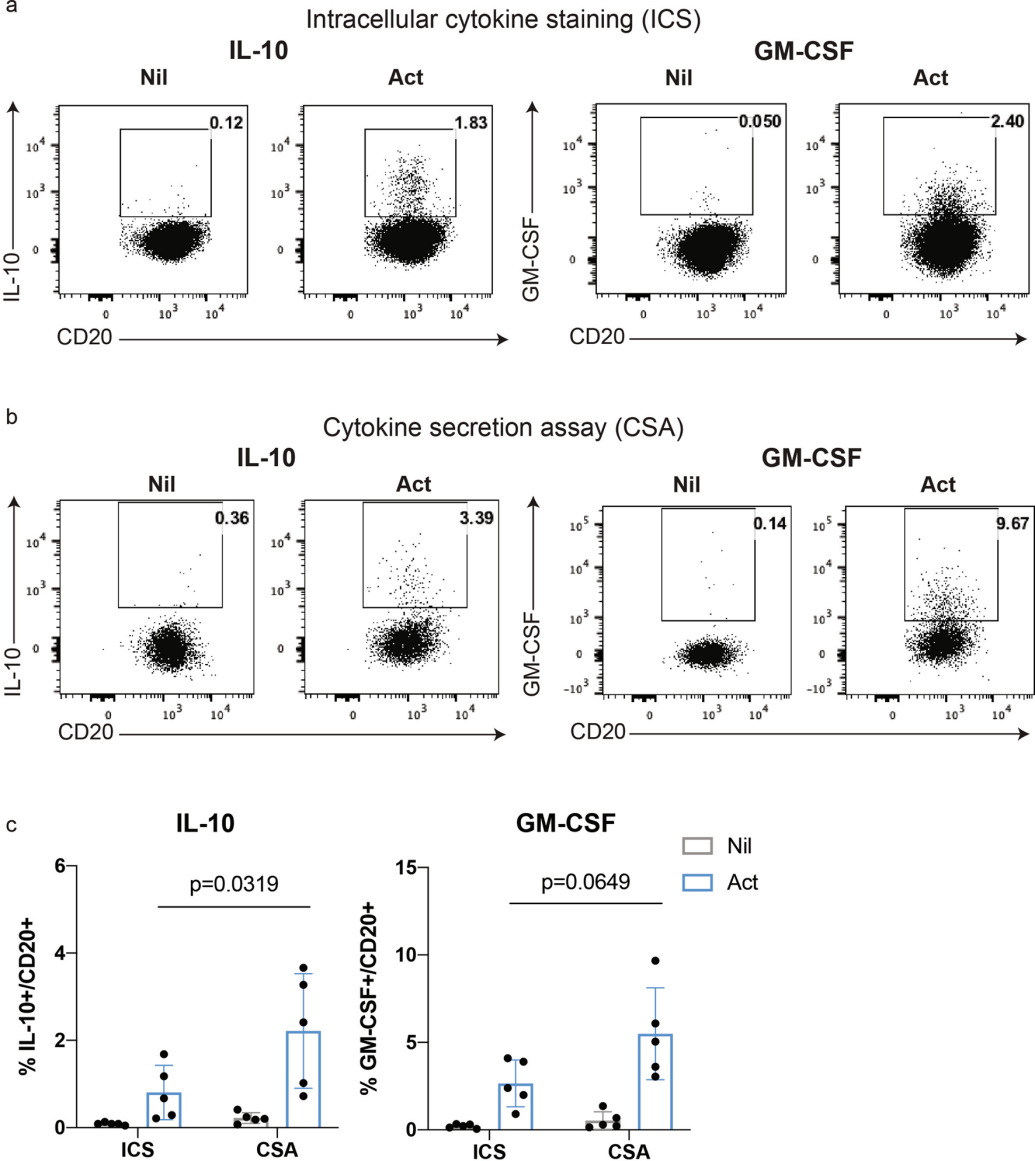
Detection of low frequency cytokine-producing human B cells using intracellular cytokine staining or the cytokine-secretion assay. Human B cells, isolated from peripheral blood mononuclear cells (PBMC) using CD19-microbeads (purity routinely confirmed by flow cytometry as > 98%), were left unstimulated (Nil) or briefly activated (Act) with PMA (20 ng/ml) and ionomycin (500 ng/ml) for 4 h before assessing IL-10 and GM-CSF expression by intracellular cytokine staining (ICS) or using the cytokine-secretion assay (CSA). Representative FACS profiles are shown for ICS (**a**) and CSA (**b**), with results of n = 5 independent experiments summarized in **(c)**. Data shown are the mean ± SD. Statistical analysis carried out with two-way ANOVA.

We first isolated highly purified (> 98%) B cells from peripheral blood mononuclear cells (PBMC) and then assessed, in parallel, the ability to detect individual, low-frequency cytokine-producing human B cells, using either intracellular cytokine staining (ICS) or the cytokine secretion assay (CSA). IL-10^+^ and GM-CSF^+^ B cells, known to be expressed by relatively low frequencies of human peripheral blood B cells^41^, can be detected following brief (4 h) stimulation with PMA and ionomycin, using either ICS (Fig. 2a) or CSA (Fig. 2b). The CSA was on par with, if not better than, ICS for the detection of either of these low frequency IL-10^+^ (CSA: 2.22 $\pm$ 0.59% vs*.* ICS: 0.81 $\pm$ 0.28%, *p* = 0.0319) or GM-CSF^+^ (CSA: 5.49 $\pm$ 1.17% vs*.* ICS: 2.65 $\pm$ 0.60%, *p* = 0.0649) B cells (Fig. 2c).

### Sorting purified and functional low-frequency cytokine-secreting B cells using CSA

**Figure 3:**
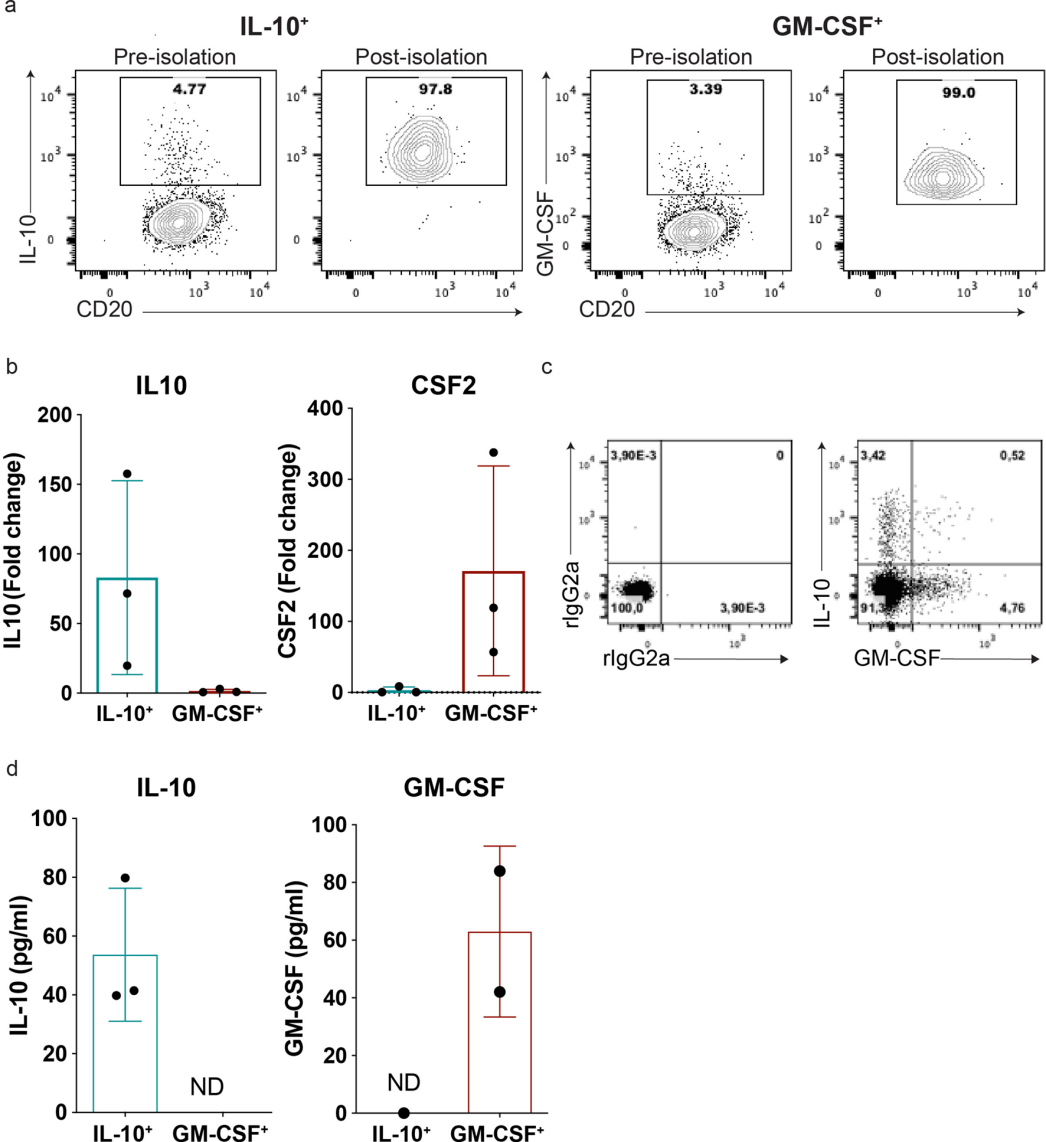
Validation of the cytokine secretion assay as an approach for isolating viable and functional low-frequency cytokine-defined human B cells. Purified human B cells were activated with PMA and ionomycin for 4 h prior to detecting the expression of either IL-10 or GM-CSF using the cytokine secretion assay. (**a**) Representative cytokine-secretion assay (CSA) FACS profiles prior to and following sorting of either IL-10^+^ or GM-CSF^+^ human B cells (BD FACSAria). (**b**) Expression levels for IL10 (left) and CSF2 (right) measured by qPCR in freshly CSA-captured and sorted IL-10^+^ and GM-CSF^+^ B cells (n = 3). (**c**) Ex vivo concurrent intracellular cytokine staining (ICS) confirms the previously reported near mutually-exclusive expression of IL-10 and GM-CSF by human B cells. Parallel staining with appropriate isotype control antibodies (rIgG2a) is included to account for background staining. (**d**) Levels of IL-10 and GM-CSF secreted by viable CSA-captured and sorted IL-10^+^ and GM-CSF^+^ B cells; 5,000 cells/well were rested overnight and re-stimulated with PMA and ionomycin for 12 h (n = 3). Data shown are the mean ± SD. Statistical analysis carried out with paired Student’s t-test. ND, non-detectable.

We next assessed whether the CSA could be used to sort and purify viable and functional low frequency cytokine-defined human B cell subpopulations. Following brief stimulation of purified human B cells, and parallel application of the CSA for either IL-10^+^ or GM-CSF^+^ B cells, fluorescence-activated cell sorting (FACS) was used to isolate the cytokine-producing B cells. Flow cytometry following sorting confirmed enrichment of > 95% (Fig. 3a). qPCR analysis demonstrated that IL10 and CSF2 expression was strikingly and reciprocally enriched in the IL-10^+^ and GM-CSF^+^ CSA-sorted B cells respectively (Fig. 3b). This data is in keeping with the near reciprocal expression of these cytokines observed when total B cells are co-stained using intracellular cytokine staining (Fig. 3c) as previously reported^41^. We next demonstrated that the CSA sorted cells were able to respond to subsequent re-stimulation, with secretion of either IL-10 or GM-CSF confirmed by ELISA in culture supernatants of the respectively sorted sub-populations (Fig. 3d). These experiments demonstrated that functional, highly-enriched low-frequency cytokine-producing B cells can be isolated using CSA and flow cytometry, and integrated into downstream applications such as gene expression analysis or in vitro cell culture.

### Simultaneous isolation of two low frequency cytokine-secreting human B cells

**Figure 4:**
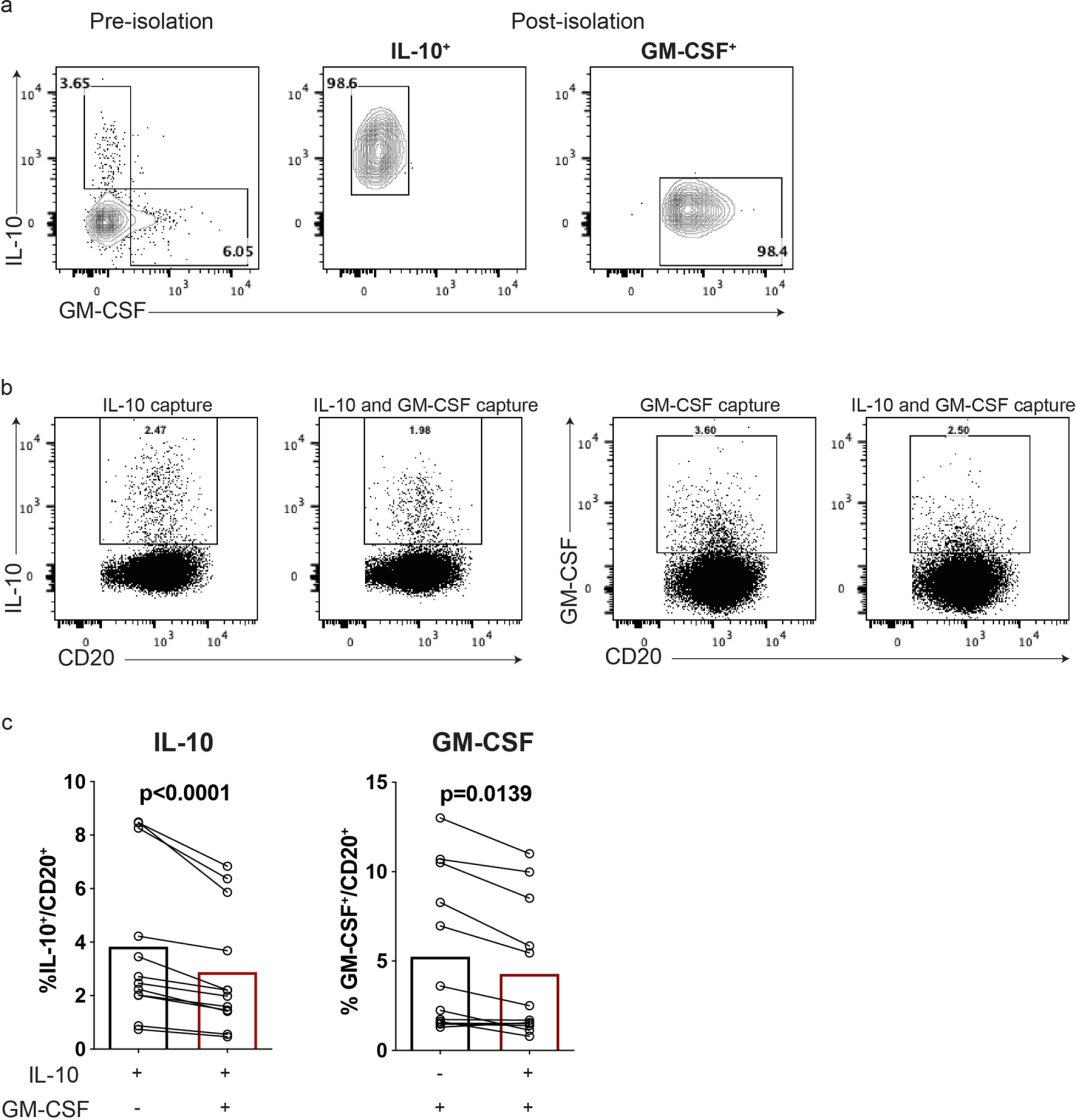
Concurrent isolation of two low-frequency cytokine-secreting B-cell subpopulations using the cytokine secretion assay. Human B cells were activated with PMA (20 ng/ml) and ionomycin (500 ng/ml) for 4 h before carrying out the cytokine secretion assay. (**a**) A representative staining of IL-10^+^ and GM-CSF^+^ B cells, prior to and following isolation using simultaneous dual cytokine-capture. The frequencies of live IL-10^+^ and GM-CSF^+^ cells were examined following ex-vivo B cell stimulation and either single cytokine capture (IL-10 or GM-CSF alone), or dual cytokine capture (both IL-10 and GM-CSF simultaneously); (**b**) staining from representative experiment; (**c**) summary of n = 12 independent experiments. Statistical analysis carried out with paired Student’s t-test.

Concurrent detection and isolation of multiple low frequency-cytokine-secreting cell sub-populations would allow for more efficient use of limited cell numbers often obtained from rare and/or limited clinical samples. We therefore next sought to adapt the CSA-Flow approach by multiplexing the capture reagents for concurrent detection and isolation of two low-frequency cytokine-expressing (IL-10^+^ and GM-CSF^+^) B cell subpopulations. We found that the multiplexing successfully yielded highly purified cytokine-defined cell-subpopulations (Fig. 4a). Compared with single cytokine-captured B cells, however, the presence of a second capture reagent resulted in a reduction in the frequencies of IL-10^+^ (single-capture: 3.83 $\pm$ 0.84% vs*.* double-capture: 2.88 $\pm$ 0.65%, *p* < 0.0001) and GM-CSF^+^ (single-capture: 5.25 $\pm$ 1.26% vs. double-capture: 4.28 $\pm$ 1.08%, *p* = 0.0139) B cells (Fig. 4b,c). Broad titration of the capture reagents did not overcome this limitation (data not shown). We conclude that detection and isolation of two, highly purified, viable low-frequency cytokine-secreting B cells are feasible using multiplexed CSA-Flow, though can result in reduced frequencies of the individual subpopulations.

### Concurrent detection of three cytokine-secreting B cell subpopulations

**Figure 5:**
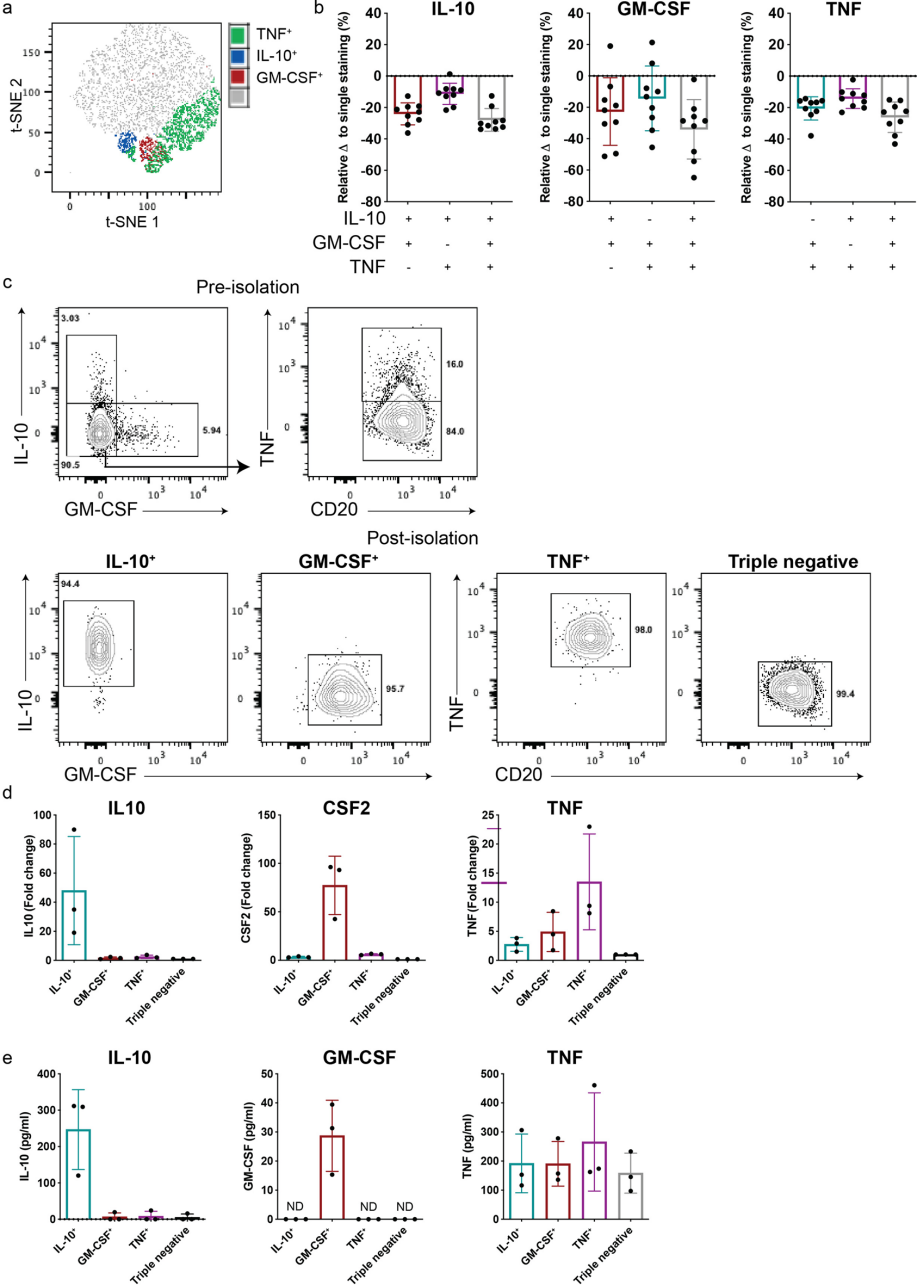
Simultaneous detection and isolation of three cytokine-secreting B cell subpopulations using the cytokine secretion assay. (**a**) Concurrent detection of three cytokine-secreting B cell populations in one sample visualized on t-SNE plot. (**b**) Relative change in the frequency of IL-10, GM-CSF and TNF in samples stained with different combinations of capture and detection reagents, as compared to individual staining (n = 9). (**c**) A representative staining of IL-10^+^, GM-CSF^+^ and TNF^+^ B cells, prior to and following isolation using simultaneous triple cytokine-capture. Triple-negative (IL-10^–^GM-CSF^–^TNF^-^) B cells were also sorted as a form of negative-control. (**d**) Expression levels for IL10 (left), CSF2 (middle) and TNF (right) were measured by qPCR in freshly CSA-captured and sorted B cells (n = 3). (**e**) Sorted B cell sub-populations were rested overnight and re-stimulated with PMA and ionomycin for 12 h. Levels of secreted cytokines were assessed by ELISA. Data shown are the mean ± SD.

We considered that concurrent isolation of low- and high-frequency cytokine-secreting cells may also be of interest. TNF is expressed by a high proportion of circulating human B cells as compared to GM-CSF or IL-10^27, 30, 32, 41^. After validating the detection and isolation of single-captured TNF^+^ B cells using CSA (Fig. S1), we integrated TNF capture into our multiparametric CSA to test the feasibility of simultaneous detection and isolation of IL-10^+^, GM-CSF^+^ and TNF^+^ B cells (t-SNE plot; Fig. 5a). To better understand assay performance, we evaluated the impact of simultaneous co-capture of two or three cytokines on the detection of individual cytokine-secreting B cells. We found that combined double-detection of the high-frequency (TNF^+^) subpopulation with either of the low frequency (IL-10^+^ or GM-CSF^+^) subpopulations, generally resulted in lesser decreases in yields of the low-frequency sub-populations than the decreased yields observed with combined detection of the two low-frequency cytokine secreting sub-populations (Fig. 5b). While triple-capture tended to result in somewhat greater decreases in yields of each of the three cytokine-defined populations (compared to single- or double-capture; Fig. 5b), the IL-10^+^, GM-CSF^+^ and TNF^+^ B cell fractions isolated by flow cytometry exhibited excellent purities (Fig. 5c) and viabilities (Fig. S2). Further, the isolated IL-10^+^ and GM-CSF^+^ B cells again exhibited the expected reciprocal enrichment for their corresponding cytokines by both qPCR (Fig. 5d) and as secreted cytokines measured by ELISA following further activation (Fig. 5e). In keeping with our prior work assessing co-expression of multiple B cell cytokines by intracellular cytokine staining^41^, we observed that TNF was expressed (both at the RNA level using qPCR and as secreted protein measured by ELISA) by a broad range of cytokine-sorted B cells, including those expressing IL-10^+^or GM-CSF^+^ (Fig. 5d,e). Overall, we describe a successful approach for simultaneous detection, and high-purity isolation, of both low- and high- frequency cytokine-defined B cell subsets using a combined CSA and flow cytometry approach (CSA-Flow) and provide useful insights on the impact of multiplexing different cytokine-capture reagents on signal detection, which will guide users in their particular applications.

## Discussion

In this study, we demonstrate the utility of the cytokine secretion assay combined with flow cytometry (CSA-Flow) in isolating viable, low- and high-frequency cytokine secreting B cells, both individually or concurrently. Isolated cell fractions were highly purified, exhibited excellent viability, and could be integrated into downstream applications such as analysis of gene expression or further in vitro activation/cell culture. The assay relies on sequential antibody-based cell surface labelling, thereby preserving cell integrity and minimizing cell loss^34, 35^. These features, along with the ability to isolate viable and functional low frequency cytokine-defined cells, encapsulate the main competitive advantage of this assay over traditional intracellular cytokine-staining or flow fluorescent in situ hybridization^19–25^.

Assay multiplexing is attractive for maximizing the output of potentially limited clinical sample volumes, and has been previously used with CSA to both isolate and distinguish distinct cytokine-secreting immune cells^42, 43^. Our data suggests that cell purities and viabilities are excellent upon multiplexing but the downside can be variable loss of signal. We speculate that signal loss during assay multiplexing was due to saturation of CD45, the cell surface anchor for the bispecific capture reagents^35^, which may impact on assay sensitivity. Reagent titration is important in optimizing signal to noise for each of the different multiplexed cytokines (general guidelines on flow-cytometry antibody titration have been discussed in recent publications^21, 44, 45^), though this does not fully mitigate partial loss of signal. While this can impact yields of the isolated subpopulations, their purities (including of the concurrently isolated low-frequency populations) following the multiplexed CSA was nonetheless excellent, making them amenable to highly sensitive interrogation. We note relatively broad variation in the magnitude of cytokine expression by the isolated subpopulations, both at the RNA-expression and secreted-protein levels which, in addition to the expected inter-individual variability, may also reflect cellular-level heterogeneity^46^ which could be of interest to elucidate.

CSA-Flow can be seamlessly integrated into multi-parametric FACS panels as part of complex cell-sorting strategies^44, 47^, as the anti-cytokine antibodies can be pre-mixed with other cell surface antibodies and simultaneously applied to cells. Prior work with CSA has included pre-enrichment of cytokine-secreting immune cells using magnetic-based isolation^34,48,49^ which can facilitate the subsequent study of particularly rare populations, and additional applications have included studies of cytokine detection kinetics^42^, cellular plasticity of cytokine expression^43,50^ and interrogation of antigen-specific cytokine-expressing cells^34,49^. We envision that CSA-Flow can also be integrated in the workflow for next-generation and single-cell technologies. This would enable analysis of molecular pathways and transcriptional networks of cells secreting different cytokines, as well as analyses of cells secreting the same cytokine, in different contexts. As an example of the latter, distinct subsets of IL-17-expressing T cells have been shown to harbor homeostatic versus pathologic properties^51,52^. Further cellular state diversity could be gleaned from combining CSA-Flow, antigen receptor sequencing and single-cell RNA-seq, as it would allow to draw relationships between antigenic specificity and cell transcriptome among seemingly homogeneous functional cellular profiles^3,4^, extending prior antigen-specific CSA applications.

In summary, we present a method for the simultaneous isolation of viable low- and high-frequency cytokine-secreting human B cells using CSA-Flow, and highlight strengths and limitations of this approach. Application of this approach should provide users with an opportunity to simultaneously and more fully elucidate the biology of distinct cytokine-defined cell subpopulations, with in-depth analysis and further functional characterization predicated on isolation of highly purified and viable cells from limited clinical samples.

## Materials and methods

### Cell Isolation and culture

Fresh blood was obtained from healthy individuals recruited from the Montreal Neurological Institute and Hospital (MNI/H), McGill University and University of Pennsylvania. All subjects provided an informed consent as approved by the corresponding institutional ethics review boards. The study was approved by and carried out in accordance with the guidelines and regulations of the Institutional Review Board of the Montreal Neurological Institute (McGill University) and University of Pennsylvania and in accordance with the Declaration of Helsinki. Peripheral blood mononuclear cells (PBMC) were isolated from whole blood using Ficoll-Paque density gradient centrifugation (GE Healthcare) and a strict standardized protocol^53^. B cells were selected from PBMC using CD19 microbeads (Miltenyi Biotec) according to manufacturer’s recommendations. Typical purities routinely assessed by flow cytometry were > 98%. B cells were cultured in serum-free X-Vivo medium (Lonza) and plated in flat-bottom 24 well plate at 2 × 10^6^/well in a total volume of 1500 μl of medium. Cells were activated with phorbol 12-myristate 13-acetate (PMA; 20 ng/ml, Sigma-Aldrich) and Ionomycin (500 ng/ml, Sigma-Aldrich) for 4 h. In the case of intracellular cytokine staining (ICS), Golgi stop (Monensin, BD Biosciences) was added to cells at the start of stimulation.

### Intracellular cytokine staining

Intracellular cytokine staining (ICS) of human B cells was performed as previously described^41^. Briefly, cells were stained with LIVE/DEAD fixable Aqua dead cell stain (Thermo Fisher Scientific) for 20 min on ice following which cell-surface marker staining was performed using mouse anti-human CD20 (BD Biosciences; clone: 2H7) and mouse anti-human CD3 (BD Biosciences; clone: UCHT1). Cells were then fixed and permeabilized using fixation/permeabilization buffer (BD Biosciences). Rat anti-human GM-CSF (clone: BVD2-21C11), rat anti-human IL-10 (clone: JES3-19F1) and mouse anti-human TNF (clone: MAb11) antibodies (BD Biosciences) or matching isotype controls were added and cells were incubated for 30 min on ice. Cells were washed and resuspended in FACS buffer (PBS/1%FCS) until analysis on a FACS LSR Fortessa (BD Biosciences).

### Cytokine secretion assay (CSA)

For the cytokine secretion assay, cells were harvested and washed once in ice-cold MACS buffer (PBS/2 mM EDTA/5%BSA). B cells were then resuspended in serum-free X-Vivo medium and labelled with capture antibodies for GM-CSF (final dilution 1:20), IL-10 (final dilution 1:10) or TNF-alpha (final dilution 1:10) (Miltenyi Biotec) for 10 min on ice. Cells were further diluted in pre-warmed medium at 1 × 10^5^ cells/ml and incubated while rotating (using a MACS rotor) at 37° C/5% CO_2_ for 45 min. B cells were then placed on ice for 10 min to terminate the secretion phase. Cells were subsequently washed twice in ice-cold MACS buffer before labelling with detection antibodies for GM-CSF (final dilution 1:20), IL-10 (final dilution 1:10) or TNF (final dilution 1:40) along with mouse anti-human CD20 (clone: 2H7) and mouse anti-human CD3 (clone: UCHT1) antibodies. We employed the anti-human TNF antibody (clone: MAb11; BD Biosciences) as a detection antibody. B cells were washed once in ice-cold MACS buffer and analyzed on BD LSRFortessa (BD Biosciences). Alternatively, cytokine-secreting B cells were sorted on BD FACSAria (BD Biosciences). Data analysis was performed with FlowJo Software (TreeStar).

### Enzyme-linked immunosorbent assay (ELISA)

Cytokine levels in cultured supernatants were measured by ELISA kits (BD Biosciences or Thermo Fisher Scientific) following manufacturer’s protocols.

### Quantification of mRNA expression by quantitative, real-time polymerase chain reaction

Total RNA extraction was performed using RNeasy Plus Micro kit (Qiagen, Valencia, CA) following manufacturer’s protocols. The RNA was stored -80C and used to generate single-stranded cDNA in a standard reverse transcription (RT) reaction using high-capacity cDNA reverse transcription kit with RNase inhibitor (Thermo Fisher Scientific). Analysis of gene expression was performed using the following TaqMan probes: CSF2 *(Hs00929873_m1),* IL10 *(Hs00961622_m1)* and TNF *(Hs01113624_g1). 18 s (Hs03003631_g1)* was used as a housekeeping gene. Fold change calculations were performed using the –ΔΔCT method.

### Statistics

All values are expressed as mean $\pm$ SD. Student’s paired t-test or two-way ANOVA with a Tukey post-hoc test was used for statistical comparisons between two groups. GraphPad Prism 8 was used to perform all the statistical analyses. P values of 0.05 or less were considered statistically significant.

## Supplementary information

Supplementary Legends.

Supplementary Information 1.

Supplementary Information 2.

## Data Availability

The datasets generated during and/or analysed during the current study are available from the corresponding author on reasonable request.
