# Supplementary material for: Multiplexed detection and isolation of viable low-frequency cytokine-secreting human B cells using cytokine secretion assay and flow cytometry (CSA-Flow)
Source: Sci Rep. 2020 Sep 9;10:14823. doi: 10.1038/s41598-020-71750-z (PMC7481209; doi:10.1038/s41598-020-71750-z)
Supplement: Supplementary file 1 — Supplementary Legends. [file 41598_2020_71750_MOESM1_ESM.docx]

**Multiplexed detection and isolation of viable low-frequency cytokine-secreting human B cells using cytokine secretion assay and flow cytometry (CSA-Flow)**

Ayman Rezk^1,2^, Rui Li^1,2^ and Amit Bar-Or^1,2,3, §^

^1^ Center for Neuroinflammation and Experimental Therapeutics (CNET) and Department of Neurology, Perelman School of Medicine, University of Pennsylvania, Philadelphia, PA, USA

^2^Neuroimmunology Unit, Department of Neurology and Neurosurgery, Montreal Neurological Institute, McGill University, Montreal, Quebec, Canada

^3^Division of Neurology, Children’s Hospital of Philadelphia, Perelman School of Medicine, University of Pennsylvania, PA, USA

^§^ Corresponding Author:

Prof. Amit Bar-Or MD, FRCPC

Melissa and Paul Anderson President’s Distinguished Professor

Director, Center for Neuroinflammation and Neurotherapeutics

Chief, Multiple Sclerosis Division, Department of Neurology

Perelman School of Medicine, University of Pennsylvania

Adjunct Professor of Neurology, Montreal Neurological Institute, McGill University

Perelman Center for Advanced Medicine (PCAM)

South Pavilion, 7^th^ Floor, Rm 763

3400 Civic Center Blvd, Philadelphia, PA 19104, USA

[amitbar@pennmedicine.upenn.edu](mailto:amitbar@pennmedicine.upenn.edu)

**Figure legends**

**Supplementary figure 1. Detection and isolation of viable TNF-secreting B cells using the cytokine secretion assay.**

Purified human B cells were left unstimulated or briefly activated with PMA (200ng/ml) and ionomycin (500ng/ml) for 4 hours before assessing TNF expression by ICS or CSA. **(a)** Representative FACS profiles are shown for ICS (left) or CSA (right). **(b)** The frequency of TNF^+^ B cells was examined following the brief activation and single cytokine capture in four independent experiments. **(c)** Expression levels for TNF measured by qPCR in freshly sorted TNF^+^ or TNF^-^ CSA-captured B cells; represented as relative change to TNF^-^ (n=3). Data shown are the mean +/- SD. Statistical analysis carried out with paired Student’s t-test.

**Supplementary figure 2. Viability of three cytokine-secreting B cell sub-populations simultaneously isolated using the cytokine secretion assay.**

Purified human B cells were stimulated with PMA (200ng/ml) and ionomycin (500ng/ml) for 4 hours prior to concurrent CSA-isolation of IL-10^+^, GM-CSF^+^ and TNF^+^ cells, as well as triple negative cells (IL-10^-^-GM-CSF^-^-TNF^-^). Cell viability was assessed using Annexin V and PI on the sorted **(a)** IL-10^+^, **(b)** GM-CSF^+^, **(c)** TNF^+^ and **(d)** triple negative (IL-10^-^-GM-CSF^-^-TNF^-^). Representative of two independent experiments.
