# Supplementary figures and images for: Multiplexed detection and isolation of viable low-frequency cytokine-secreting human B cells using cytokine secretion assay and flow cytometry (CSA-Flow)
Source: Sci Rep. 2020 Sep 9;10:14823. doi: 10.1038/s41598-020-71750-z (PMC7481209; doi:10.1038/s41598-020-71750-z)

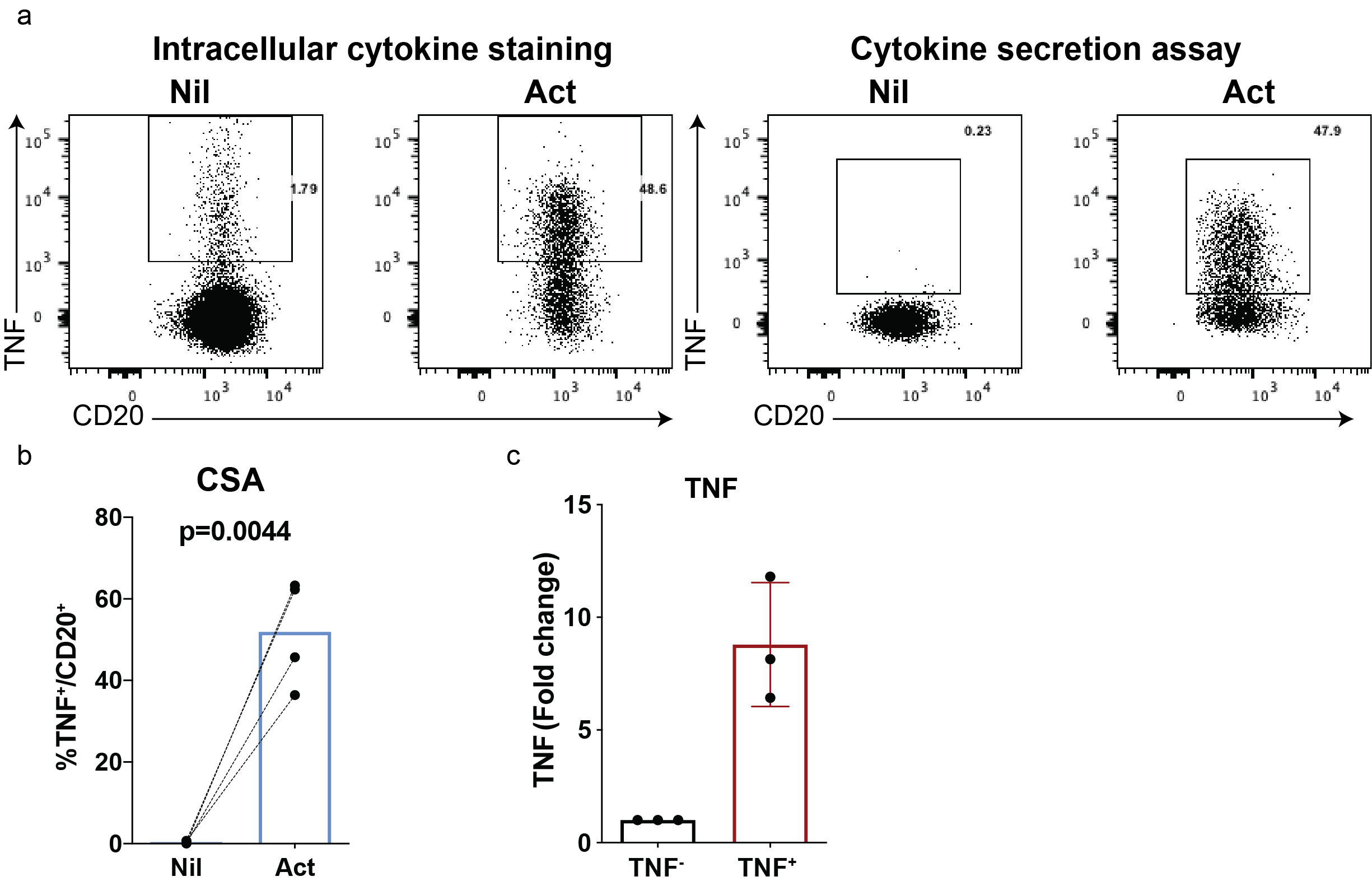

Supplement: Supplementary file 2 — Supplementary Information 1. [file 41598_2020_71750_MOESM2_ESM.jpg]

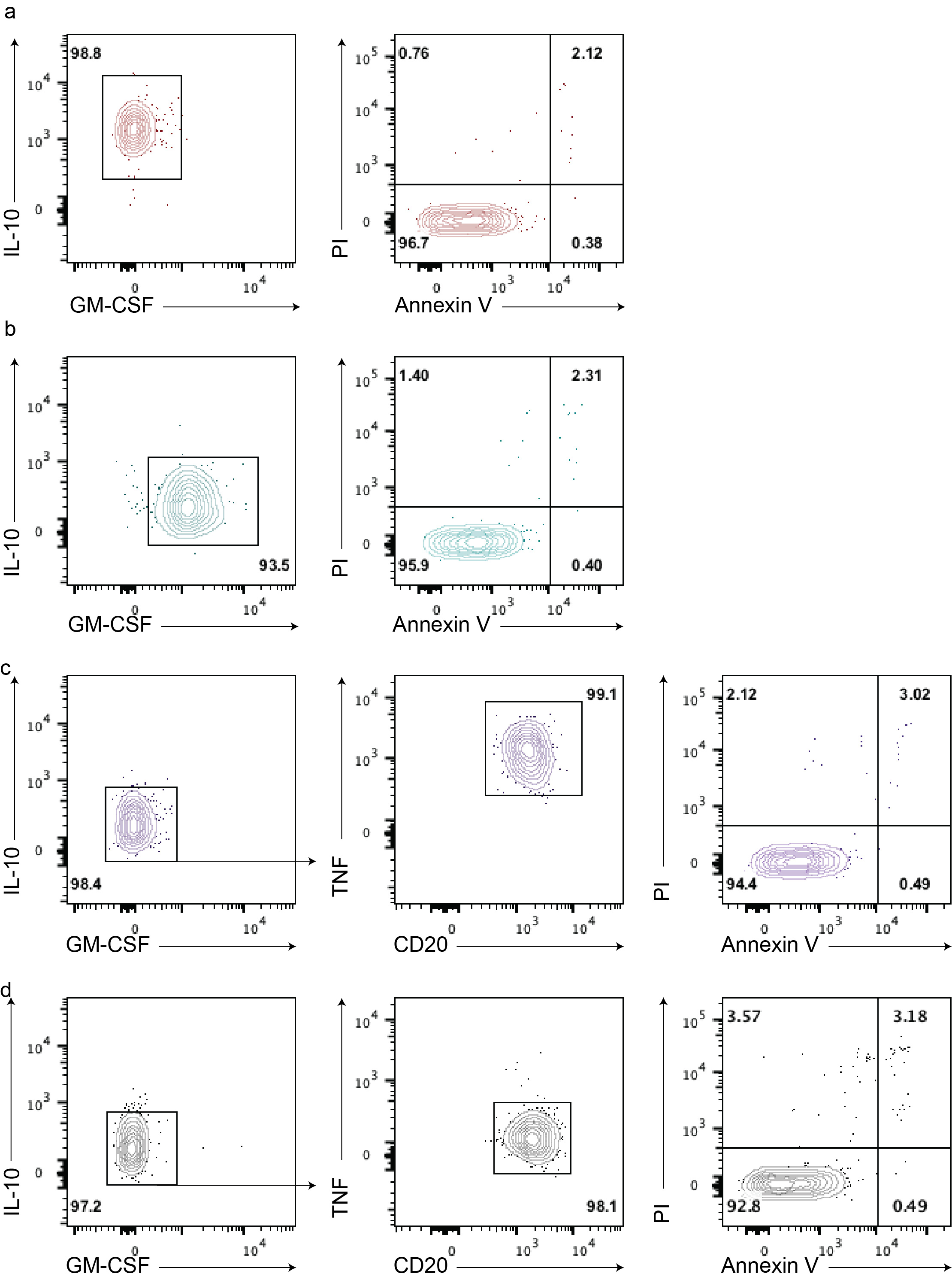

Supplement: Supplementary file 3 — Supplementary Information 2. [file 41598_2020_71750_MOESM3_ESM.jpg]
